# Short-term clinical outcomes of balloon-assisted thrombectomy for acute middle cerebral artery occlusive

**DOI:** 10.3389/fneur.2026.1796509

**Published:** 2026-04-15

**Authors:** Bin Liao, Xiuling Yang, Zhesi Xing, Guoshun Li, Zichong Liang, Huiquan Deng, Zhaobang Chen, Jiasheng Zhao, Huanlun He, Wenfei Liang, Jingling Zhu, Yu Ding, Qiuxing He, Jingyi Chen, Weimin Ning

**Affiliations:** Department of Neurology, Dongguan Hospital of Guangzhou University of Chinese Medicine, Dongguan, China

**Keywords:** acute ischemic stroke, balloon-assisted thrombectomy, endovascular therapy, middle cerebral artery occlusion, short-term outcomes

## Abstract

**Objective:**

This study aimed to evaluate the short-term efficacy and safety of balloon-assisted thrombectomy (BAT) for acute middle cerebral artery (MCA) occlusion caused by thrombus superimposed on intracranial atherosclerotic stenosis via a retrospective analysis.

**Methods:**

From January 2023 to July 2025, 341 consecutive stroke patients who underwent endovascular therapy at the Department of Neurology, Dongguan Hospital of Traditional Chinese Medicine were initially screened. According to predefined inclusion and exclusion criteria, 45 patients were selected for the final analysis. All included patients received vessel recanalization using the BAT technique. Baseline demographic and clinical data were recorded. Short-term outcomes, procedural metrics, safety endpoints, and economic/technical aspects were assessed.

**Results:**

A total of 45 patients treated with BAT were analyzed. Procedural outcomes demonstrated a first-pass mTICI ≥2b recanalization rate of 75.56%, and final mTICI 2b-3 was achieved in 100% of patients. Median puncture-to-recanalization time was 35 min (23.50–51.00). Clinical and safety outcomes at 90 days showed a modified Rankin Scale (mRS) of 0–2 (independent) in 73.33% and an optimal EQ-5D-5L health state in 42.22%; all-cause mortality was 2.22%. Logistic regression analysis indicated that puncture-to-reperfusion time was the only independent predictor of a favorable 90-day clinical outcome (mRS ≤ 2) after BAT (OR = 0.878, 95% CI: 0.793–0.973, *p* < 0.01). Economically, BAT reduced resource utilization by streamlining procedural steps and decreasing intraoperative stent deployment.

**Conclusion:**

Balloon-assisted thrombectomy for acute MCA occlusion due to thrombus on stenotic lesions achieves efficient vessel recanalization with low complication rates and favorable short-term functional outcomes, while offering economic advantages. BAT may represent a preferred endovascular strategy for this subset of complex lesions.

## 1 Introduction

Acute large vessel occlusions (LVOs) are a severe subtype of ischemic stroke (IS) (1), associating with high rates of disability and mortality (2), posing substantial threats to patient survival and quality of life. The middle cerebral artery (MCA), as the principal intracranial vessel of the anterior circulation (3), is the most commonly involved site in anterior-circulation LVOs, accounting for over 60% of cases (4). Patients with MCA occlusion typically present with more severe neurological deficits and worse outcomes.

Endovascular thrombectomy (EVT) has been established by multiple randomized controlled trials as the standard of care for acute anterior-circulation LVOs and significantly improves long-term outcomes (5–7). However, complex lesion morphology—such as vessel tortuosity, underlying intracranial atherosclerotic stenosis with superimposed thrombus—remains a challenge for EVT (8, 9). Stent-retriever thrombectomy often requires repeated maneuvers and device adjustments, which prolong procedure time and increase the risk of thrombus fragmentation, endothelial injury, and distal embolization (10–12). Aspiration-only techniques may have limited clot-engagement capability in tortuous anatomy, failing to achieve complete thrombus removal and risking emboli to new territories (ENT) or the same distal territory (EDT) (13), thereby worsening clinical prognosis.

Balloon-assisted thrombectomy (BAT) was originally developed in percutaneous coronary intervention (PCI) to facilitate device passage through tortuous or stenotic vessels without causing vessel trauma (14). The technique involves partially extruding and inflating a balloon distal to the guide catheter tip at nominal pressure to allow smooth device advancement over the guidewire while minimizing mechanical injury to the vessel wall. Recent case reports and small series have suggested the feasibility of BAT in neurointervention (15), offering a potential approach for treating complex intracranial occlusions.

Compared with conventional EVT techniques, BAT integrates proximal balloon-mediated flow control, catheter aspiration, and thrombectomy devices to provide several theoretical advantages: the inflated balloon at the catheter tip can reduce antegrade flow and promote reverse flow control, lowering the risk of thrombus fragmentation and distal embolization (16); the streamlined balloon–catheter assembly reduces a “cutting” effect on the vessel intima, potentially improving reperfusion quality (17–19); and the simplified workflow may decrease intraoperative reliance on adjunctive stent implantation, offering both clinical and economic benefits. Nevertheless, clinical evidence for the BAT in MCA occlusion is limited, and standardized procedural guidance is lacking—particularly regarding the optimal timing of balloon inflation and pressure settings when treating stenosis with superimposed thrombus.

In this retrospective study, we focus on patients with acute MCA occlusion to evaluate whether BAT can achieve high first-pass reperfusion rates in complex anatomy and to assess 90-day functional outcomes following use of this technique. The results aim to provide targeted evidence to refine thrombectomy strategies and support standardization of BAT for intracranial stenotic thrombotic lesions.

## 2 Methods

### 2.1 Research design and participants

This study followed the principles of the Helsinki Declaration and was approved by the Institutional Review Board of Dongguan Hospital (No. PJ [2025] 165). Informed consent was waived since this study involved a retrospective analysis of existing databases.

A single-center, retrospective, observational cohort study was conducted at Dongguan Hospital of Traditional Chinese Medicine (Dongguan, China). A total of 341 patients diagnosed with acute ischemic stroke with large vessel occlusion and treated with endovascular therapy between January 5, 2023, and July 31, 2025, were included in this study. We retrospectively reviewed the inpatient conditions of the patients, focusing on the use of the BAT technique after acute intracranial arterial stenosis and thrombosis formation, which was our primary observational outcome.

*Inclusion criteria*: Following the standards outlined in the 2019 American Stroke Association guidelines for early management of acute ischemic stroke patients (20). Head CT scans ruled out intracranial hemorrhagic lesions; pre-stroke mRS score of 0-1; meeting clinical diagnostic criteria for acute ischemic stroke with confirmation of responsible large vessel occlusion by neuroimaging (CT/MRI); time window for anterior circulation occlusion endovascular treatment defined as follows: patients in the hyperacute phase (onset ≤6 h) were directly included, while patients in the subacute or progressive stroke phase (onset 6–24 h or >24 h) with imaging findings consistent with the inclusion criteria set in the DAWN and DEFUSE-3 studies were included (20); use of BAT for vascular recanalization; lesion in the large vessel segment of the middle cerebral artery; age ≥18 years.

*Exclusion criteria*: Extensive brain parenchymal damage; concurrent active hemorrhagic disorders or significant bleeding tendencies; multi-organ failure; age ≥90 years; data inadequacy.

### 2.2 Data collection

Based on literature review and clinical experience, 29 clinically relevant variables were selected for ease of acquisition and relevance to the study. The following data were systematically extracted from electronic medical records: (1) Demographic characteristics: age, gender, medical history (smoking history, alcohol history, hypertension, diabetes, hyperlipidemia, atrial fibrillation, coronary artery atherosclerotic heart disease). (2) Clinical features: length of hospital stay (days), vital signs (admission systolic blood pressure), thrombolysis status, neurologic deficit assessment using the National Institutes of Health Stroke Scale (NIHSS) (21), which consists of 15 items with a total score ranging from 0 to 42. A score of <5 indicates minor deficits, 5–15 indicates moderate neurologic deficit, and ≥16 indicates severe neurologic deficit. mRS score (22): using the modified Rankin Scale to standardize the naming of global disability outcomes, including 7 levels (0–6 points), with 0–2 indicating good function and 3–6 indicating dependence. ASPECTS score (Alberta Stroke Program Early CT Score) (23): a quantitative scoring system based on non-contrast head CT scans for rapid assessment of early ischemic changes in the middle cerebral artery supply area, with a total score of 0–10, ≥6 indicating a small infarct core and <6 indicating a large infarct core. (3) Etiology classification: atherosclerosis-related stroke, cardiogenic stroke, other determined causes, undetermined etiology. (4) Surgery-related: Clot Burden Score (CBS) (24): ranging from 0 to 10, describing the extent of thrombus; a score of 10 is normal, 0 indicates complete multi-segment vessel occlusion, scores range from 0–6, 7–10 (25); collateral vessel status assessed using the American Society of Interventional and Therapeutic Neuroradiology (ASITN)/Society of Interventional Radiology (SIR) system (26): a total of 4 levels, 0–2 indicating poor collateral circulation, 3-4 indicating good collateral circulation. Puncture-to-reperfusion time, guide-to-reperfusion time, adjuvant therapy, and vascular tortuosity. (5) Lesion characteristics: isolated stenosis, stenosis with thrombus, arterial dissection. (6) Complications: hemorrhagic transformation after infarction, subarachnoid hemorrhage. (7) EQ-5D-5L score (27): The EQ-5D-5L assesses whether patients have no, some, or extreme problems in five dimensions of health: mobility, self-care, usual activities, pain, and anxiety, which are combined into a five-digit number describing the respondent’s health state. A score of “11111” indicates no problems, a score of “55555” indicates extreme problems in all five dimensions, while other scores indicate some problems (28).

### 2.3 Endovascular treatment

All patients in this group did not receive antiplatelet therapy before the operation. Following a preoperative cranial computed tomography (CT) scan to exclude intracranial hemorrhage, patients were administered a continuous intravenous infusion of tirofiban 24–48 h preoperatively. A follow-up cranial CT scan confirmed the absence of bleeding. Twenty-four hours after the completion of the intravenous infusion, patients started oral aspirin 100 mg combined with clopidogrel 75 mg, which was continued until a 3-month postoperative follow-up. For patients undergoing stent implantation procedures, the dual antiplatelet therapy mentioned above was continued for 3 months before adjusting the medication regimen based on the patient’s condition.

Patients were placed in the supine position, and those scheduled for endovascular treatment received general anesthesia. Before the start of the interventional procedure, routine disinfection and draping of the bilateral groin areas were performed. Local anesthesia with 1% lidocaine was administered, and the right femoral artery was punctured using the Seldinger technique. After successful puncture, an 8F arterial sheath was inserted, and unfractionated heparin was infused at a standard dose of 100 U/kg, with a bridging dose of 50 U/kg for the patient. A diagnostic catheter was used to confirm the occlusion of the MCA. Subsequently, an 8F guiding catheter was inserted and advanced to the C1 segment of the Internal Carotid Artery (ICA) under wire guidance. The specific target vessel was the MCA main trunk. The guiding catheter was positioned appropriately to establish vascular access. Preoperative device selection was based on the diameter of the middle cerebral artery (MCA). Balloon catheters measuring 2.0 * 15 mm and 2.25 * 15 mm were preferentially selected and matched with 5F and 6F aspiration catheters, respectively. The balloon diameter was chosen to be slightly larger than that of the aspiration catheter.

**Figure 1:**
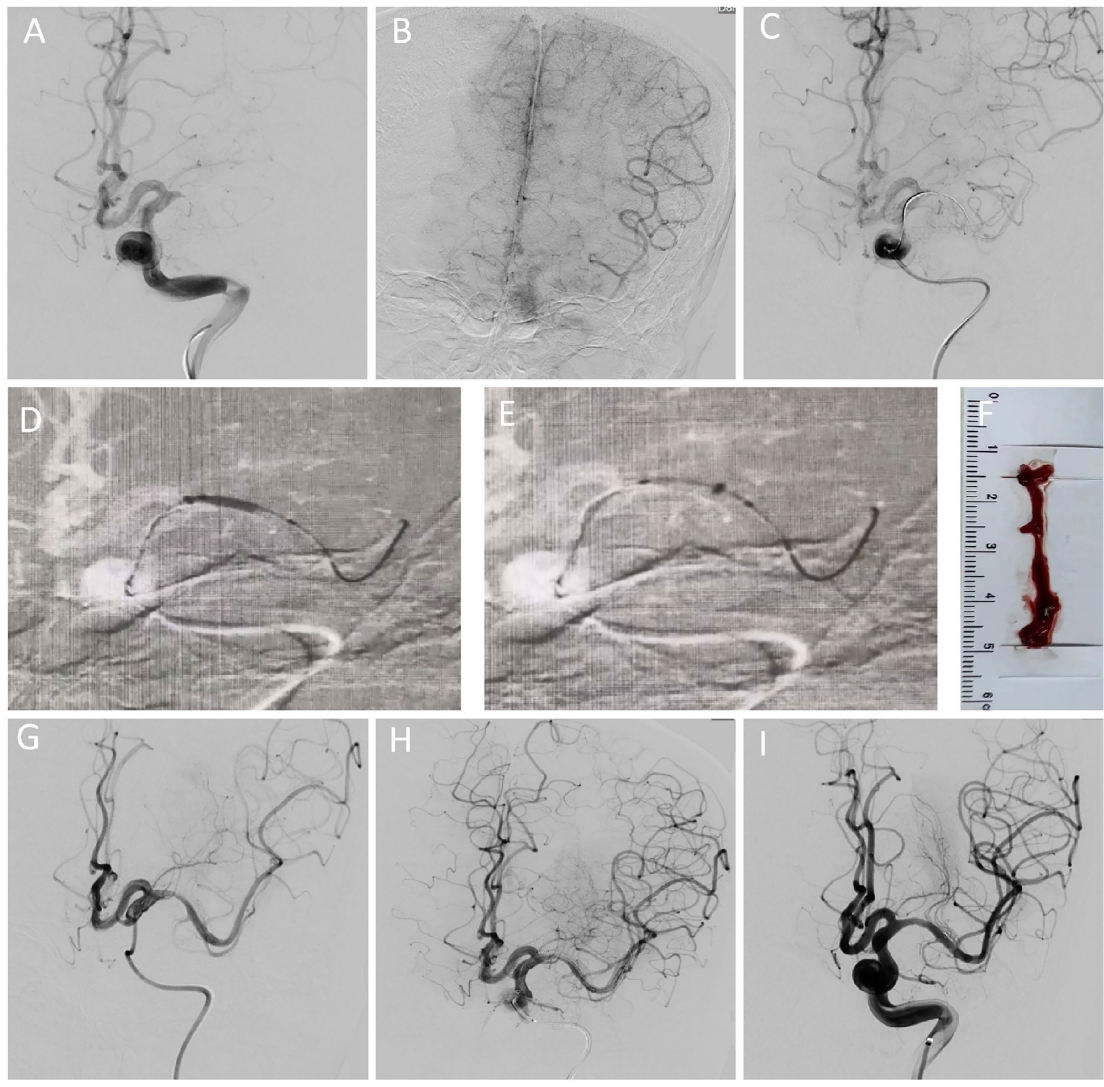
Patient presented with sudden right-sided limb weakness and loss of consciousness, with the last known normal time being 5 h prior to admission. Initial head CT from an outside facility showed no signs of hemorrhage. **(A,B)** Cerebral angiography revealed occlusion of the left middle cerebral artery with a tapered appearance and partial meningeal collateral circulation compensation in the late arterial phase. **(C)** Confirmation of distal true lumen patency after passage through the lesion site using a microguidewire and microcatheter, with a positive initial pass and a significant thrombus seen post-narrowing. **(D,E)** Cross-passage performed at the stenotic lesion site using the BAT technique. **(F,G)** Significant thrombus clearance was achieved at the distal lesion end during aspiration; follow-up imaging showed severe narrowing at the M1 segment of the middle cerebral artery. **(H,I)** Effective vessel remodeling following balloon dilation for proximal stenosis; an Enterprise 4 * 23 mm stent was implanted in the M1 segment of the middle cerebral artery to achieve mTICI 3 reperfusion. Post-BAT and mechanical thrombectomy, no signs of intracranial hemorrhage or extensive cerebral infarction were observed.

Under fluoroscopic guidance, a 0.014in * 200 cm microguidewire (Synchro 14) was advanced across the lesion. A microcatheter was used to assess the nature of the lesion via the first-pass effect. The balloon was initially inflated slowly at a low pressure of 3-4 atm to dilate the stenotic segment and maintained for 30 s to achieve luminal remodeling. Following balloon dilation, a balloon anchoring technique was employed. The aspiration catheter was advanced to engage the proximal end of the balloon, forming an integrated and smooth configuration to reduce shear stress on the vessel wall. After docking with the proximal balloon segment, the balloon was gradually deflated while carefully advancing the aspiration catheter until it reached one-third of the balloon’s distal portion. Continuous fluoroscopic monitoring was maintained throughout the procedure to prevent vascular injury. Once the balloon was fully deflated, the aspiration catheter was synchronously advanced beyond the occluded segment to perform thrombus aspiration. Continuous negative pressure was maintained during aspiration to ensure effective thrombectomy. During slow withdrawal of the aspiration catheter, negative pressure was maintained across the stenotic segment and gradually released while retracting, thereby removing residual thrombus and plaque debris. In this technique, approximately half of the balloon remained within the aspiration catheter and the other half outside. The balloon was inflated at the stenotic segment to achieve angioplasty while the aspiration catheter was simultaneously advanced to the distal side of the stenosis for thrombus aspiration (Figure 1).

### 2.4 Statistical analysis

Statistical analysis was primarily conducted using SPSS software (IBM version 31.0). Descriptive statistics were performed on 45 participants. For continuous variables, the Kolmogorov–Smirnov test (K-S test) was employed. If *p* > 0.05, the data were considered to follow a normal distribution and presented as mean ± standard deviation (SD). Otherwise, for non-normally distributed data, the median and interquartile range were used. Categorical data were summarized using frequencies (*n*) and percentages (%).

## 3 Results

**Figure 2:**
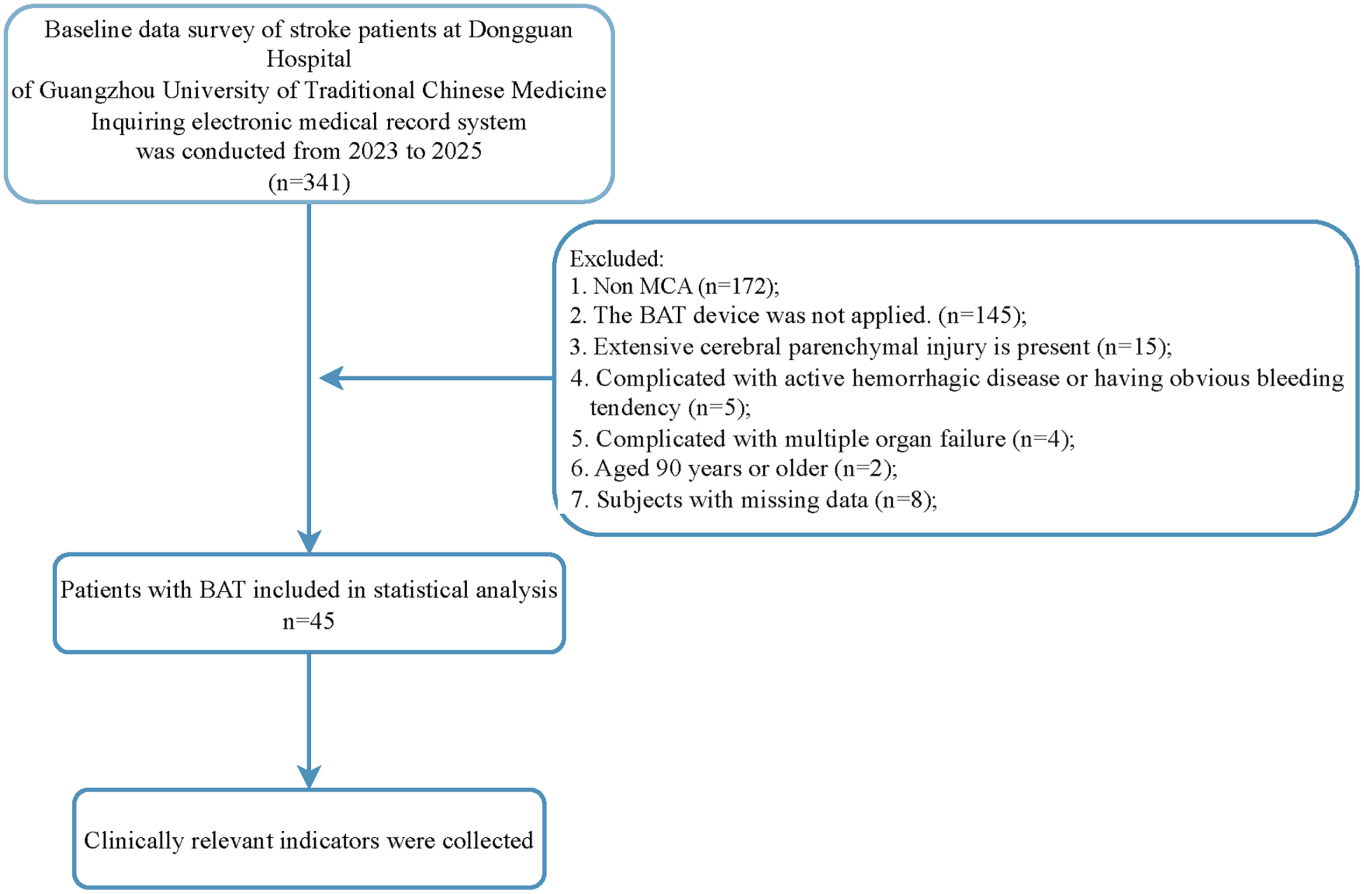
Flow chart of BAT for MCA occlusion.

Refer to Figure 2 for an illustration of the study workflow. Among the initially screened 341 eligible patients, 296 were excluded. Ultimately, 45 patients who underwent BAT treatment were included in the analysis, and relevant clinical data were collected.

During the thrombectomy procedures conducted in 341 cases throughout the study, a total of 45 patients diagnosed with acute ischemic stroke accompanied by significant stenosis of the middle cerebral artery were included. All these patients underwent recanalization therapy using the BAT technique. The study cohort predominantly consisted of male patients, accounting for 80%, with an average age of 58.82 years. In terms of medical history, a significant proportion had a history of hypertension, dyslipidemia, and smoking, accounted for 75.56, 42.22 and 53.33%, respectively. Looking at the distribution of etiologies, atherosclerotic stroke accounted for the majority at 75.56%, while cardioembolic stroke accounted for 2.22%, as shown in Table 1.

**Table 1:** Demographic characteristics and etiological classification of patients.

Characters	Values
Demographics	
Gender, male, *n* (%)	36 (80%)
Age, years, mean ± SD	58.82 ± 13.03
Medical history	
Hypertension, *n* (%)	34 (75.56%)
Diabetes, *n* (%)	11 (24.44%)
Dyslipidemia	19 (42.22%)
Atrial fibrillation, *n* (%)	2 (4.44%)
Coronary artery disease, *n* (%)	1 (2.22%)
Smoking, *n* (%)	24 (53.33%)
Alcohol drinking, *n* (%)	10 (22.22%)

Etiology	Values
Atherosclerosis-related stroke, *n* (%)	40 (88.89%)
Cardiogenic stroke, *n* (%)	1 (2.22%)
Other determined cause, *n* (%)	3 (6.67%)
Undetermined etiology, *n* (%)	1 (2.22%)

Upon admission, the average systolic blood pressure of the patients was 146.49 mmHg. In terms of admission NIHSS scores, 64.44% of strokes scored between 5–15 points, while 26.67% had mRS scores of 0–2 points and, and 73.33% scored between 3–5 points. Patients with ASPECTS scores ≥6 accounted for 73.33%, as shown in Table 2.

**Table 2:** IVT, intravenous thrombolysis, using alteplase.

Clinical features		Values
Length of hospital stay, day, median (IQR)		11 (8–14)
Systolic pressure, mmHg, mean ± SD		146.49 ± 21.72
Admission NIHSS, score, *n* (%)	0–4	13 (28.89%)
	5–15	29 (64.44%)
	16–42	3 (6.67%)
Admission mRS, score, *n* (%)	0–2	12 (26.67%)
	3–5	33 (73.33%)
ASPECTS, *n* (%)	0–5	12 (26.67%)
	6–10	33 (73.33%)

To achieve intracranial arterial recanalization, all patients underwent the BAT. Clot Burden Score (CBS) is a core indicator quantifying thrombus extent and complexity, with scores correlating with reperfusion difficulty and clinical outcomes (25, 29); in Table 3, 66.67% scored 0–6 points. ASITN/SIR grading can predict reperfusion success, final infarct size, and clinical outcomes (30), with 88.89% graded 0–2. Following a comprehensive assessment by two experienced neurosurgeons, the median Puncture-to-Reperfusion Time (PRT) was 35 min. Arterial dissection was present in 8.89% of cases. During BAT treatment, 28.99% of patients received adjunctive therapies such as stent placement at stenotic sites. The presence of vascular tortuosity post-stenosis was 24.44%, consistent with the anatomy of the MCA. 71.11% of patients had stenosis accompanied by distal thrombus formation, while 20% presented with isolated stenosis.

**Table 3:** HT, hemorrhagic transformation; SAH, subarachnoid hemorrhage.

Outcome and Procedural Characteristics		Values
Surgical indicator		
CBS, *n* (%)	0–6	30 (66.67%)
	7–10	15 (33.33%)
ASITN/SIR, *n* (%)	0–2	40 (88.89%)
	3-4	5 (11.11%)
Puncture-reperfusion, minutes, median (IQR)		35 (23.50–51.00)
Auxiliary treatment, *n* (%)		13 (28.99%)
Vascular curvature, *n* (%)		11 (24.44%)
Lesion category		
Simple stenosis, *n* (%)		9 (20.00%)
Stenosis with thrombus, *n* (%)		32 (71.11%)
Arterial dissection, *n* (%)		4 (8.89%)
Complications		
HT, *n* (%)		4 (8.89%)
SAH, *n* (%)		1 (2.22%)
Clinical outcome		
First-pass mTICI 2b-3, *n* (%)		34 (75.56%)
Final mTICI 2b-3, *n* (%)		45 (100.00%)
90 day mRS, *n* (%)	0–2	33 (73.33%)
	3–6	12 (26.67%)
Mortality		1 (2.22%)
EQ-5D-5L		
Any other health state		25 (55.56%)
11111		19 (42.22%)
55555		1 (2.22%)

Among clinical complications, the incidence of intracranial hemorrhage was 8.89%, and subarachnoid hemorrhage was 2.22%. Those achieving initial reperfusion of mTICI 2b-3 grade accounted for75.56%, with all patients eventually achieving mTICI 2b-3 reperfusion. At 90 days, 73.33% of patients had mRS scores of 0–2, with an all-cause mortality rate of 2.22% during follow-up. In the EQ-5D-5L health state assessment, 42.22% of patients scored “11111” (no health problems across dimensions), 55.56% were in other health states, and only 2.22% scored “55555” (severe health problems across dimensions).

**Figure 3:**
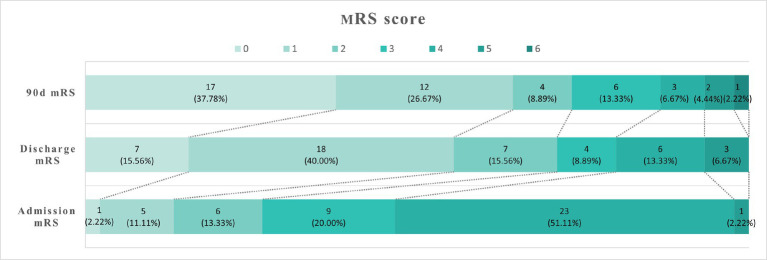
They were the number and proportion of patients with mRS scores at admission, discharge, and 90 days after surgery, respectively.

To evaluate the short-term efficacy of BAT, the mRS was used to assess patients at 90 days after BAT treatment, with follow-up conducted via outpatient revisit and telephone interview (Figure 3). The number of individuals with mRS scores of 0–2 at 90 days, as depicted in Table 3, increased compared to admission and discharge. Patients showed significant recovery post BAT procedure, with one mortality case noted, likely attributed to patient complications or the possibility of treatment abandonment by family members upon discharge.

This study constructed univariate and multivariate logistic regression models to evaluate predictors of favorable functional outcomes (mRS ≤ 2) at 90 days after BAT (Table 4). Systolic blood pressure, puncture-to-reperfusion time, first-pass mTICI 2b-3 recanalization, and HT after infarction were significant predictors of favorable functional outcomes (mRS ≤ 2) at 90 days after BAT in the univariate analysis. However, in the multivariate analysis, puncture-to-reperfusion time was the only independent predictor of favorable functional outcome at 90 days after BAT (OR = 0.878, 95% CI: 0.793–0.973, *p* = 0.013). This indicates that for every 1-min increase in puncture-to-reperfusion time, the odds of achieving a favorable functional outcome decreased by 12.2%. In contrast, systolic blood pressure (OR = 1.040, 95% CI: 0.979–1.106, *p* = 0.204) and first-pass mTICI 2b/3 recanalization (OR = 0.807, 95% CI: 0.060–10.872, *p* = 0.871) did not reach statistical significance in the multivariate model and therefore could not be considered independent predictors.

**Table 4:** Predictors of favorable clinical outcome (mRS ≤ 2) at 90 days after BAT.

Variable	Univariate OR (95% CI)	*p*-value	Multivariate OR (95% CI)	*p*-value
Systolic pressure	1.038 (0.998–1.078)	0.06	1.040 (0.979–1.106)	0.204
Puncture-reperfusion	0.886 (0.809–0.970)	0.009	0.878 (0.793–0.973)	**0.013**
First-pass mTICI 2b-3	5.6 (1.277–24.564)	0.022	0.808 (0.060–10.872)	0.871
HT	0.094 (0.009–1.014)	0.051		

Variables with *p* < 0.1 in the univariate logistic analysis were included in the collinearity diagnostics. Hemorrhagic transformation (HT) showed a variance inflation factor (VIF) >5 and was therefore excluded from the multivariate logistic regression analysis. In the multivariate logistic regression model, *p* < 0.05 was considered statistically significant, as indicated by the bold values.

## 4 Discussion

Acute MCA occlusion is a critical subtype of ischemic stroke with high disability and mortality rates (2). Timely and effective vascular recanalization is crucial for improving neurological outcomes and reducing adverse events. In clinical practice, MCA lesions often present with complexities such as vascular tortuosity, calcified plaques, or near-complete occlusions (31, 32), making endovascular treatment daunting. Particularly in cases with stenosis combined with high thrombus burden, traditional mechanical thrombectomy has limitations. Stent retrievers prolong procedure times and increase the risk of thrombus fragmentation, vascular injury, and distal embolization (33–35). Pure aspiration alone may struggle to completely clear thrombi within tortuous vessels and can lead to ENT or EDT. While mechanical thrombectomy remains the frontline treatment for large vessel occlusion due to intracranial atherosclerosis (ICAS-LVO) (36), there is room for further improvement in optimizing distal thrombus clearance efficiency, reducing reocclusion rates at stenotic sites, and minimizing procedure-related complications.

With the continuous evolution of endovascular treatment techniques, it has been increasingly utilized in patients with proximal intracranial stenosis accompanied by distal thrombus formation. Li et al. (15) have demonstrated the feasibility of BAT in managing such complex lesions. However, most prior studies are small case series that focus mainly on basic clinical outcomes and procedural success, without patient health-related quality of life. In addition, their study populations are not precisely defined, limiting the applicability of their conclusions to specific anatomical subgroups. In contrast, this study specifically focused on patients with acute MCA-M1 occlusion characterized by proximal stenosis accompanied by distal thrombus formation, excluding other subtypes of intracranial arterial occlusion. Beyond evaluating procedural characteristics and conventional outcomes, we also incorporated health-related quality of life for the first time. By reducing the need for intraoperative stent implantation and device exchanges, our approach also lowered medical resource utilization, providing an economic rationale for the broader clinical adoption of BAT.

In this study, all cases of acute ischemic stroke involving large vessels were related to the middle cerebral artery, with most cases showing stenosis in the MCA M1 segment accompanied by distal thrombus formation. Traditional mechanical thrombectomy poses challenges in treating such conditions. However, the use of the BAT technique significantly reduces surgical risks. The Clot Burden Score (CBS), which quantifies thrombus extent and complexity, is crucial in assessing recanalization difficulty and clinical prognosis (25, 29). The proportion scoring 0–6 is higher than in a typical acute middle cerebral artery infarction (37), indicating potential challenges in achieving efficient recanalization and a higher risk of distal embolization. ASITN/SIR grades can predict reperfusion success, final infarct size, and clinical outcomes (30). The high proportion of grades 0–2 suggests predominant poor collateral circulation, redistributing blood flow through leptomeningeal anastomoses, alleviating infarct expansion and post-treatment hemorrhage (26).

The Puncture-to-Reperfusion Time (PRT) for BAT treatment was shorter compared to median times reported in multicenter trials (38), attributed to the simplified operational process and reduced time for instrument exchange. By integrating balloon and aspiration catheters’ functions, BAT efficiently opens vessels, especially in cases with high thrombus burden and poor collateral circulation. This study’s analysis of prognostic factors further indirectly confirmed that the application of BAT is an independent protective factor for improving 90 day functional outcomes. In the multivariate logistic regression analysis, puncture-to-reperfusion time was the only independent predictor of a favorable clinical outcome (mRS ≤ 2) after BAT. By achieving rapid and effective reperfusion of the ischemic territory, shorter puncture-to-reperfusion time contributes to better functional recovery. The data from this study on BAT show a first-pass success rate of 75.56% in cases with high thrombus load and poor collateral circulation, achieving complete mTICI 2b-3 reperfusion in all cases. This high first-pass success rate surpasses traditional mechanical thrombectomy techniques (5, 39, 40), highlighting the efficiency of BAT in achieving recanalization in complex stroke scenarios. Its technical advantage lies in optimized design, which allows precise positioning with the assistance of a semi-compliant balloon, facilitating rapid traversal through narrow and tortuous vessel segments (41), avoiding thrombus displacement or fragmentation associated with repeated adjustments in conventional thrombectomy procedures. By temporarily occluding proximal blood flow with the balloon, the risk of thrombus escape during retrieval is reduced (42), enabling direct and efficient thrombus capture and removal. Moreover, improved vessel compliance from balloon dilation provides a smoother path for subsequent thrombus clearance, reducing the probability of multiple retrieval attempts. This “one-step” technical characteristic not only shortens the critical Puncture-to-Reperfusion Time but also increases the rate of successful initial complete recanalization of occluded vessels, reducing mechanical damage to fragile vessels, compensating for inadequate collateral circulation, and minimizing the conversion of ischemic penumbra to infarction. This sets a foundation for rapid cerebral perfusion recovery and improved neurological outcomes in patients with poor collateral circulation.

The predominant clinical complications observed in this study were bleeding-related events, with no significant occurrences of vessel injury or distal embolization related to the procedure. A good functional outcome with mRS scores at 90 days accounted for 73.33%, demonstrating a clear advantage over reported data from traditional mechanical thrombectomy techniques (43). The EQ-5D-5L results further confirm the clinical benefits of BAT. Patients showed a favorable distribution of health-related quality of life post-treatment, with 42.22% no health issues across dimensions, while only 2.22% were in the worst healthy state. The EQ-5D-5L scale, as an internationally recognized tool for assessing health-related quality of life (44), quantifies post-stroke patients’ physiological function and psychological status. The scores directly correlate with post-stroke neurological recovery, providing a more objective reflection of the treatment’s value in improving patients’ actual quality of life. These data substantiate the good short-term efficacy and safety of Balloon-assisted Tracking (BAT) in treating complex large vessel occlusive strokes. Due to BAT’s shorter Puncture-to-Reperfusion Time and higher rate of initial successful recanalization, the duration of cerebral ischemia is reduced, maximizing the survival of ischemic penumbral brain tissue and creating favorable conditions for neurological function recovery in patients. Additionally, the differences in health-related quality of life scores across dimensions can aid in developing personalized rehabilitation plans for post-stroke follow-up patients, leading to a higher rate of good functional outcomes, a lower incidence of complications, and improved quality of life.

It should be noted that bleeding-related complications still occurred in a certain proportion in this study. Although not significantly higher than safety thresholds reported in existing guidelines, further risk reduction is warranted through optimizing surgical procedures (such as precise control of balloon inflation pressure, avoiding excessive recanalization), strict adherence to patient selection criteria, among other means. This study lacked a direct control group and did not include cases treated with conventional stent retriever thrombectomy or aspiration alone, additionally, given the limitations of sample size and follow-up duration in this study, the long-term safety and efficacy of BAT technology require further validation through large-sample, multicenter prospective studies.

The main innovations of this study lie in its precise focus on the study population and detailed recording of clinical parameters. In comparison to previous studies covering various types of vascular occlusions, this study specifically targeted MCA occlusion patients, eliminating interference from other intracranial vascular occlusion subtypes, making the study conclusions more targeted and clinically relevant. In terms of data collection, beyond routine recording of recanalization rates and prognostic scores, this study systematically collected key procedural parameters such as Puncture-to-Reperfusion Time, and provides more detailed data support for analyzing optimization points in the treatment process and standardizing operational procedures. By incorporating the EQ-5D-5L scale into the post-BAT treatment efficacy assessment system for the first time, establishing a causal relationship between technical advantages, neurological function recovery, and quality of life improvement, and providing a basis for developing individualized rehabilitation plans. Furthermore, BAT technology simplifies operational steps, reduces device exchanges, integrates balloon and aspiration catheter functions, and efficiently manages lesions with stenosis and thrombi, effectively opening vessels. By reducing the use of stents during procedures, BAT lowers medical costs compared to traditional stent retrievers, demonstrating greater cost-effectiveness and clinical applicability.

## 5 Conclusion

This study confirms that BAT treatment for acute middle cerebral artery occlusive stroke has good short-term recanalization efficacy and safety, effectively improving patients’ short-term prognosis. Furthermore, this technique is faster and more cost-effective compared to conventional thrombectomy procedures.

## 6 Limitations

There are certain limitations to this study. Firstly, the study design is retrospective, inevitably leading to selection bias. There may be an imbalance in the clinical baseline data of included patients, and the relatively limited sample size might affect the generalizability of the results. Secondly, this study only focuses on short-term efficacy, and long-term follow-up is needed to assess the impact of BAT on patients’ long-term quality of life. Lastly, there was no control group directly comparing BAT with other thrombectomy techniques. Future studies could conduct large-scale controlled research to further validate the efficacy of BAT, extend the follow-up period, refine long-term prognosis data, and provide more comprehensive evidence for the clinical application of this technique.

## Data Availability

The original contributions presented in the study are included in the article/supplementary material, further inquiries can be directed to the corresponding authors.
